# Arkansas Oil Stone

**Published:** 1847-03

**Authors:** 


*Arkansas Oil Stone.*—This is truly a remarkable stone, and to none is it more serviceable than to the *dentist;* and it is particularly fortunate, that one of the proprietors is, himself, a practitioner of dental surgery, and a man eminently capable of appreciating the value of any article that can add perfection to the art.

The gentleman alluded to is Dr. A. Vancamp, of Nashville, Tennessee. Dr. V. has prepared this stone in every variety of shape, not only for the purpose of sharpening dental instruments, for which it is immeasurably preferable to any other oil stone, but also for finishing stoppings in the mouth. For this purpose nothing can exceed it, and by having it prepared of proper shapes it may be used with advantage in any position which a plug may be situated.

This stone on account of its great hardness, (the property which gives it its peculiar merit,) is necessarily expensive, when wrought, as is now done, expressly for the use of the dentist. We saw, a few days since, a quantity from Dr. V's manufactory, cut into the shape of the various dental files, many pieces sufficiently thin to be used between front teeth in finishing stoppings.

The profession are greatly indebted to Dr. Vancamp for this contribution to their conveniences, for finishing their operations beautifully.—*Syracuse Ed.*

